# Incidence and classification of pediatric diffuse parenchymal lung diseases in Germany

**DOI:** 10.1186/1750-1172-4-26

**Published:** 2009-12-12

**Authors:** Matthias Griese, Melanie Haug, Frank Brasch, Achim Freihorst, Peter Lohse, Rüdiger von Kries, Theodor Zimmermann, Dominik Hartl

**Affiliations:** 1 Dr von Haunersches Kinderspital, University of Munich, Lindwurmstr 4a, D-80337 Munich, Germany; 2 Institut für Pathologie, Klinikum Bremen-Mitte gGmbH, St-Jürgen-Str 1, 28177 Bremen, Germany; 3 Kinderklinik, Ostalb Klinikum, Im Kälblesrain 1, 73430 Aalen, Germany; 4 Clinical Chemistry, University of Munich, Marchioninistr 15, 81377 München, Germany; 5 Institut für Soziale Pädiatrie und Jugendmedizin im Kinderzentrum München, Abteilung für Epidemiologie des Kindes- und Jugendalters, Heiglhofstr 63, 81377 München, Germany; 6 Universitätskinderklinik Erlangen, Loschgestr 15, 91054 Erlangen, Germany

## Abstract

**Background:**

Diffuse parenchymal lung diseases (DPLD) in children represent a rare and heterogeneous group of chronic pulmonary disorders. Despite substantial advances in genetics and pathomechanisms, these often lethal diseases are still under-diagnosed. This is due to the fact that (i) the incidence is low, and (ii) clinical presentation, (iii) disease classification and (iv) specific treatment options are largely unknown.

**Methods:**

Here we systematically assessed the incidence, the presentation, the diagnostic yield and treatments of pediatric DPLD in Germany, using the Surveillance Unit for Rare Paediatric Disorders (ESPED).

**Results:**

The incidence of DPLD was 1.32 new cases per 1 million of children per year. The majority of these children were diagnosed within the first year of life. Overall survival was 87%. Using centralized data entry and stratification tools, the patients were categorized into an advanced classification system based on diagnostic algorithms, including clinical presentations, genetics and/or histology. Combining molecular and clinical information, this survey provides an etiological overview and specific diagnostic recommendations for children with DPLD.

**Conclusions:**

Standardized surveys and systematic classifications are valuable tools for the clinical handling of children with DPLD and aim to improve the disease understanding and the prognosis of these rare detrimental lung diseases.

## Background

Diffuse parenchymal lung diseases (DPLD) in children, also called pediatric interstitial lung diseases, comprise a large and heterogeneous group of chronic pulmonary disorders [1,2]. These diseases are characterized by impaired gas exchange and radiological diffuse infiltrates and have a substantial morbidity and mortality. The major current challenges in DPLD are difficulties with a lack of confidence concerning the (i) clinical diagnosis classification and resulting (ii) therapeutic options. This is mainly due to the paucity of epidemiological data, classification standards and evidence based treatment recommendations in DPLD. The majority of reports represent single cases or small series and not much information is available on the incidence and the natural course of these diseases in pediatric patients. This results in the perpetuation of imprecise diagnoses and non-systematic management with potential significant therapeutic side effects.

Recently, several attempts have been made to overview the epidemiology and clinical outcome of children with DPLD. A national survey in the United Kingdom and Ireland collected 46 cases of children with histological proven idiopathic interstitial pneumonitis over a three years period [3]. The prevalence for children aged 0-16 years was estimated to be 3.6 cases/million; no numbers on the incidence were reported. Similarly, the task force on chronic interstitial lung diseases in immuno-competent children of the European Respiratory Society reported on a broad variety of cases. A total of 185 prevalent cases (107 with biopsy results available) were retrieved across Europe. Since the number of non-reporting centers remained unknown, no numbers on the prevalence of these diseases could be derived [4]. In these recent studies histology was used alone or, at least to a significant extent, for disease classification. However, as a comprehensive classification of the diseases was not available in these studies, these reports are of limited use for clinical diagnosis and treatment of children with DPLD.

Similarly as for the adult idiopathic interstitial pneumonias, when an international group of experts for the clinical, radiographic, and pathologic features agreed upon a consensus classification in 2002 [5] a novel classification was proposed by a working group on pediatric DPLD [6]. This classification system was primarily based on lung biopsy findings derived from 187 cases in children less than 2 years of age with DPLD collected between July 1999 and July 2004 in eleven children's hospitals in North America. This classification system represents a major step forward towards a thorough definition of pediatric cases of DPLD, because it allows a far-reaching categorization of almost all currently known DPLD entities. The resulting nomenclature dichotomizes the DPLD diseases into entities mainly prevalent in infants (A) and entities occurring at all ages (B). Each group contains 4 subgroups, i.e. diffuse developmental disorders (A1), alveolarization abnormalities (A2), specific conditions of undefined etiology (A3) and surfactant dysfunction disorders (A4). With respect to those entities occurring at all ages (B), the subgroups related to systemic diseases (B1), in immune-intact hosts (B2), in immunocompromised hosts (B3) and disorders masquerading as DPLD (B4).

The goal of this present study was to assess the incidence, classification, the clinical management and the outcome of pediatric DPLD in Germany based on the current DPLD classification system, supported by an established tool for epidemiologic studies on rare diseases, the German ESPED research unit [7].

## Methods

The survey was performed with the help of the German Surveillance Unit for Rare Paediatric Disorders (Erhebungseinheit für seltene pädiatrische Erkrankungen in Deutschland (ESPED)). This survey unit sends out monthly postcards or emails to all paediatric hospitals, usually addressing several responsible persons in each hospital, who report whether or not DPLD has been diagnosed. Between January 1, 2005 and December 31, 2006, monthly 462 cards and emails were sent out simultaneously in Germany. The overall return rate was 97% for both card and email responses. In case of a positive reply, a detailed questionnaire was sent to the reporting institution. The return rate was 70%. In cases of lacking or incompletely filled out questionnaire, information was attempted to obtain by phone. The number of children under 17 years of age under surveillance was 14 393 400.

The case definition included all cases of newly diagnosed patients with DPLD. These were defined as (i) neonates (> 36 weeks of gestation) with chronic (longer than 6 weeks) respiratory distress and disease diagnosing findings in bronchoalveolar lavage, lung biopsy, or identification of disease causing mutations in the genes for the ABCA3-transporter, surfactant protein B and C (SP-B, SP-C) and (ii) all children less than 17 years of age with chronic (more than 6 weeks) diffuse parenchymal lung disease. In particular, these disease entities included pulmonary interstitial glycogenosis, persistent tachypnoea of infancy (or neuroendocrine cell hyperplasia in infancy), alveolar proteinosis, chronic pneumonitis of infancy, follicular bronchitis or broncholitis, desquamative interstitial pneumonitis (DIP), lymphocytic interstitial pneumonia (not due to recognized immune deficiency), non-specific interstitial pneumonitis (NSIP), idiopathic pulmonary fibrosis of infancy, pulmonary hemosiderosis, sarcoidosis, hypersensitivity pneumonitis and eosinophilic pneumonias. DPLDs, secondary due to immuno deficiencies, rheumatological or oncological diseases, or a chronic graft-versus-host-reaction of the lungs following stem cell or solid organ transplantation, were excluded in this study.

The pseudonymized questionnaire asked for sex, age, age at diagnosis, other diagnoses, ethnicity, consanguinity, family history, gestational age at birth and respiratory problems in the neonatal period, pulmonary signs and symptoms, loss of weight, results of diagnosing tests (blood, biopsy, bronchoalveolar lavage, lung function, genetic tests) and therapy for the diffuse parenchymal lung disease diagnosed and was approved by the Bavarian data protection agency. In all cases in which a lung biopsy was obtained, tissue was investigated by light microscopy, including PAS, iron and bombesin stain and thereafter by electron microscopy where indicated.

The detailed information collected was discussed and verified by an independent steering group consisting of pediatric pulmonologists (MG, AF, TZ), a geneticist (PL), and a pediatric pathologist (FB). Those cases that met the inclusion criteria qualified as DPLD. These cases were included into the study.

The results are reported as individual values. For comparison of categorical variables, the Fisher exact test was used. A two sided p-value of < 0.05 was considered significant.

## Results

### Overall characteristics of the patients

**Figure 1:**
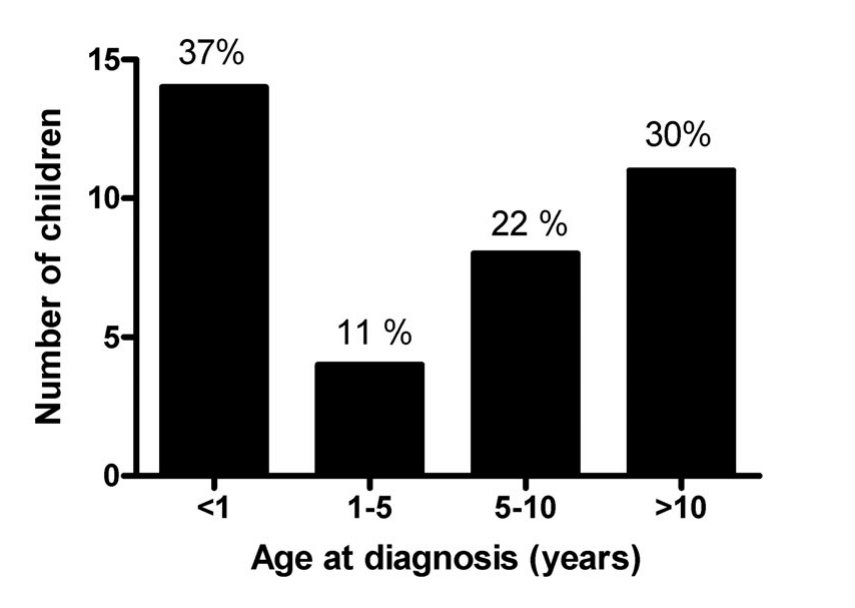
**Children with diffuse parenchymal lung diseases incident during 2005 and 2006 in Germany divided into different age groups**. Given are absolute numbers and the percentages are indicated on top of the columns.

The overall response rate was excellent and was 97%. Of the 56 cases reported, after review by the steering committee, thirty eight cases were verified as DPLD and included in the final study population (Tables 1, 2 and 3). The incidence of pediatric DPLD in Germany was calculated as 1.32 (confidence interval 0.92 to 1.81) new cases per 1 million of children per year. The female to male ratio was 1.10. The delay in diagnosis, i.e. the time between initial symptoms and the final diagnosis was on average 2 months (range 0 to 12 months). About one third of the cases manifested their disease within the first year of life (Figure 1). After the first year of life, there was a continuous increase of the proportion of children within a certain age group. Important hints for a genetic cause of the DPLD were obtained from the family history on ethnic background, consanguinity and affection of other family members; this was positive in 6 of the 12 infants with initial symptoms during the neonatal period (Tables 4 and 5).

**Table 1:** Categorization and diagnosis - Entities mainly prevalent in infants (A)

Patient No.	Interstitial lung disease	Final pulmonary diagnosis	Classifi-cation of pediatric DPLD*	Additional diagnosis	Center type	Coop-Diagnosis***	Lung biopsy, result	Genetics SFTPB, SFTPC, ABCA3
1	Related to alveolar surfactant region	SP-B deficiency, SFTPB mutations	A4		R	yes	CPI	yes
2	Related to alveolar surfactant region	SP-C deficiency, SFTPC mutation	A4	GERD	U	no	NSIP, Cholesterol pneumonitis	yes
3	Related to alveolar surfactant region	ABCA3 two mutations**, SFTPC mutation	A4	Neonatal infection with E. coli, hyperglycemia, anemia	U	yes	No	yes
4	Related to alveolar surfactant region	ABCA3 two mutations**	A4		R	yes	CPI	yes
5	Related to alveolar surfactant region	ABCA3 two mutations**	A4	Failure to thrive	U	yes	DIP	yes
6	Related to alveolar surfactant region	ABCA3 one mutation, low SP-C in lavage	A4	Partial IgA deficiency	U	yes	DIP, post infectious residuals	Yes
7	Related to alveolar surfactant region	NSIP	A4		U	no	NSIP, Cholesterol pneumonitis	n.k.
8	Related to alveolar surfactant region	PAP, juvenile, sporadic	A4		U	yes	PAP	No
9	Unclear RDS in the mature neonate	SP-C deficiency, (extremely low lavage level)	A4, possible	Partial albinism, bilateral inner ear deafness, microcephalus, hypertrophic cardiomyopathy, diaper rash, failure to thrive, dyskinesia	U	yes	no	Yes

*Classification of pediatric DPLD according to Deutsch et al 2007; entities mainly prevalent in infants (A; i.e. diffuse developmental disorders (A1), alveolarization abnormalities (A2), specific conditions of undefined etiology (A3) and surfactant dysfunction disorders (A4)). Entities occurring at all ages (B; i.e. related to systemic diseases (B1), in immune-intact hosts (B2), in immunocompromised hosts (B3) and disorders masquerading as DPLD (B4)). A4, possible; Ax and Bx denote children with the clear clinical diagnosis of pediatric DPLD, in whom however the diagnostic process was stopped prematurely.

**Two mutations means compound heterozygous or homozygous mutations in the ABCA3 gene

***Diagnosis in co-operation with German consultation network for rare lung diseases

Abbreviations: HSP, Hypersensitivity pneumonitis, NEHI, Neuroendocrine cell hyperplasia of infancy; PAP, pulmonary alveolar proteinosis; NSIP, non-specific interstitial pneumonitis; DIP, desquamative interstitial pneumonitis; R, regional hospital; U, University hospital; n.d., not determined; SFTPB or SFTPC, gene encoding surfactant protein B or C.

**Table 2:** Categorization and diagnosis - Entities mainly prevalent in infants (A), continues table 1

Patient No.	Interstitial lung disease	Final pulmonary diagnosis	Classifi-cation of pediatric DPLD*	Additional diagnosis	Center type	Coop-Diagnosis***	Lung biopsy, result	Genetics SFTPB, SFTPC, ABCA3
10	Unclear RDS in the mature neonate	Blepharo stenosis bilateral, pigmentation abnormalitis of the retina, cutis laxa, minor syndromic abnormalities, ventricle septal defect	A4, possible	VSD, hypoglycemia, choanal stenosis, blepharo stenosis	R	yes	no	yes
11	Unclear RDS in the mature neonate	n.d.	A4, possible	PDA, neonatal infection, small for date	R	yes	no	Yes
12	Unclear RDS in the mature neonate	Familial, pneumothorax	A4, possible	Hyperbilirubinemia, arterial hypotonia	U	yes	no	yes
13	Unclear RDS in the mature neonate	n.d.	A4, possible		R	no	no	yes
14	Unclear RDS in the mature neonate	Pulmonary hypertension, recurrent pneumothoraces	A4, possible	Persistant fetal circulation, acute renal failure, central disorder of muscle tone and coordination, suspected neonatal infection	U	yes	no	no
15	Unclear RDS in the mature neonate	n.d.	A4, possible		R	no	no	n.k.
16	No further categorisation	n.d.	Ax	Recurrent airway infections	R	no	no	n.k.
17	No further categorisation	n.d.	Ax	Velofacial syndrome (CATCH 22)	R	no	no	yes
18	No further categorisation	n.d.	Ax		R	no	n.k.	n.k.
19	Infant conditions of undefined etiology	NEHI	A3	GERD	U	yes	NEHI	yes
20	Infant conditions of undefined etiology	Chronic tachypnoe of infancy (CTI)	A3	Hemangioma lower lip	R	yes	no	yes
21	Infant conditions of undefined etiology	NEHI	A3	Failure to thrive	U	yes	NEHI	yes

*Classification of pediatric DPLD according to Deutsch et al 2007; entities mainly prevalent in infants (A; i.e. diffuse developmental disorders (A1), alveolarization abnormalities (A2), specific conditions of undefined etiology (A3) and surfactant dysfunction disorders (A4)). Entities occurring at all ages (B; i.e. related to systemic diseases (B1), in immune-intact hosts (B2), in immunocompromised hosts (B3) and disorders masquerading as DPLD (B4)). A4, possible; Ax and Bx denote children with the clear clinical diagnosis of pediatric DPLD, in whom however the diagnostic process was stopped prematurely.

**Two mutations means compound heterozygous or homozygous mutations in the ABCA3 gene

***Diagnosis in co-operation with German consultation network for rare lung diseases

Abbreviations: HSP, Hypersensitivity pneumonitis, NEHI, Neuroendocrine cell hyperplasia of infancy; PAP, pulmonary alveolar proteinosis; NSIP, non-specific interstitial pneumonitis; DIP, desquamative interstitial pneumonitis; R, regional hospital; U, University hospital; n.d., not determined; SFTPB or SFTPC, gene encoding surfactant protein B or C.

**Table 3:** Categorization and diagnosis - Entities occurring at all ages (B), continues tables 1 and 2

Patient No.	Interstitial lung disease	Final pulmonary diagnosis	Classifi-cation of pediatric DPLD*	Additional diagnosis	Center type	Coop-Diagnosis***	Lung biopsy, result	Genetics SFTPB, SFTPC, ABCA3
22	Related to lung vessels/heart	Idiopathic pulmonary hemosiderosis	B1	Anemia	U	no	Pulmonary hemosiderosis	no
23	Related to lung vessels/heart	Idiopathic pulmonary hemosiderosis	B1	Celiac disease	U	no	no	n.k.
24	Related to systemic disease	Sarcoidosis	B1	Familial small stature, anemia	R	no	Sarcoidosis	n.k.
25	Immune intact host	HSP	B2		U	no	HSP	n.k.
26	Immune intact host	HSP	B2		U	yes	HSP	no
27	Immune intact host	HSP	B2	Celiac disease	R	no	HSP	n.k.
28	Immune intact host	HSP	B2		U	no	no	n.k.
29	Immune intact host	HSP	B2		U	no	no	n.k.
30	Immune intact host	HSP	B2	Insufficiency of tricuspidal valve	R	no	no	n.k.
31	Immune intact host	HSP	B2		U	yes	no	n.k.
32	Immune intact host	HSP	B2		R	no	no	n.k.
33	Immune intact host	HSP	B2		R	no	no	n.k.
34	Immune intact host	HSP	B2		U	no	no	n.k.
35	Immune intact host	HSP	B2	Diabetes mellitus type I, hyperthyreosis, adipositas	R	yes	no	n.k.
36	No further categorisation	n.d.	Bx	Status following Clamydia pneumoniae pneumonia	R	no	no	n.k.
37	No further categorisation	n.d.	Bx		R	no	no	n.k.
38	No further categorisation	n.d.	Bx	Adenoids, hypertrophy of tonsils, episodes of upper airway obstruction	R	no	no	n.k.

*Classification of pediatric DPLD according to Deutsch et al 2007; entities mainly prevalent in infants (A; i.e. diffuse developmental disorders (A1), alveolarization abnormalities (A2), specific conditions of undefined etiology (A3) and surfactant dysfunction disorders (A4)). Entities occurring at all ages (B; i.e. related to systemic diseases (B1), in immune-intact hosts (B2), in immunocompromised hosts (B3) and disorders masquerading as DPLD (B4)). A4, possible; Ax and Bx denote children with the clear clinical diagnosis of pediatric DPLD, in whom however the diagnostic process was stopped prematurely.

**Two mutations means compound heterozygous or homozygous mutations in the ABCA3 gene

***Diagnosis in co-operation with German consultation network for rare lung diseases

Abbreviations: HSP, Hypersensitivity pneumonitis, NEHI, Neuroendocrine cell hyperplasia of infancy; PAP, pulmonary alveolar proteinosis; NSIP, non-specific interstitial pneumonitis; DIP, desquamative interstitial pneumonitis; R, regional hospital; U, University hospital; n.d., not determined; SFTPB or SFTPC, gene encoding surfactant protein B or C.

**Table 4:** History, symptoms, family history - Entities mainly prevalent in infants (A)

Patient No.	Gestational age (wks)	Age at diagnosis (Y)	Respiratory failure	Tachy-/Dyspnea	Chronic cough	Failure to thrive	Ethnic background	Consanguinity	Same disease in other family members
1	36*	0	yes	yes	n.k.	n.k.	Turkey	yes	yes
2	40*	6.5	yes	yes	n.k.	n.k.	Egypt	no	yes
3	40*	0	yes	yes	no	n.k.	UAE	n.k.	yes
4	38*	0	yes	yes	no	no	Syria	yes	no
5	40	2.92	yes	yes	n.k.	yes	Germany	no	no
6	40	0.75	no	yes	no	no	n.k.	n.k.	no
7	40	11.25	no	yes	n.k.	no	Japan/Poland	no	no
8	n.k.	9.08	no	yes	n.k.	yes	n.k.	n.k.	n.k.
9	37*	0.25	yes	yes	no	no	Turkey	yes	yes
10	37*	0	yes	yes	no	no	Germany	no	no
11	39*	0	yes	yes	n.k.	no	Germany	no	no
12	38*	0	yes	yes	no	no	Germany	n.k.	yes
13	40	n.k.	no	n.k.	n.k.	no	n.k.	no	no
14	38*	0	yes	yes	no	no	n.k.	n.k.	n.k.
15	40*	0.42	yes	yes	n.k.	yes	Germany	no	no
16	40	1.92	no	yes	yes	no	Germany	no	no
17	36*	0.17	no	n.k.	n.k.	yes	Germany	no	no
18	34*	0.00	yes	n.k.	n.k.	yes	n.k.	no	no
19	36	0.58	no	yes	no	no	n.k.	no	no
20	38	0.33	yes	yes	n.k.	no	Germany	no	no
21	n.k.	1.75	no	yes	n.k.	yes	Germany	n.k.	no

* Initial symptoms within the neonatal period

n.k., not known;

**Table 5:** History, symptoms, family history - Entities occurring at all ages (B), continues table 4

Patient No.	Gestational age (wks)	Age at diagnosis (Y)	Respiratory failure	Tachy-/Dyspnea	Chronic cough	Failure to thrive	Ethnic background	Consanguinity	Same disease in other family members
22	40	11	n.k.	n.k.	n.k.	yes	Turkey	yes	no
23	40	16.5	no	yes	no	no	Germany/USA	no	no
24	40	13.67	no	yes	no	no	Turkey	no	no
25	n.k.	6.17	no	yes	yes	no	Germany	no	no
26	40	10.5	no	yes	yes	no	Germany	no	no
27	36	13.5	no	yes	yes	yes	Germany	no	no
28	n.k.	5.67	no	yes	no	no	Germany	no	no
29	39	6	no	yes	yes	no	Germany	no	no
30	42	8.08	no	yes	yes	no	Germany	no	no
31	n.k.	9.42	no	yes	no	no	Germany	no	no
32	39	10.58	no	yes	no	no	Germany	no	no
33	40	11.42	no	yes	yes	no	Germany	no	no
34	40	12.58	no	yes	yes	yes	Germany	no	n.k.
35	n.k.	14.5	no	yes	yes	no	Germany	n.k.	no
36	40	13.92	no	no	no	no	Germany	no	no
37	39	n.k.	no	yes	no	no	Germany	no	no
38	n.k.	3.42	no	yes	no	no	Germany/n.k.	n.k.	yes

* Initial symptoms within the neonatal period

n.k., not known;

### Categorization into the current classification system for pediatric DPLD

**Figure 2:**
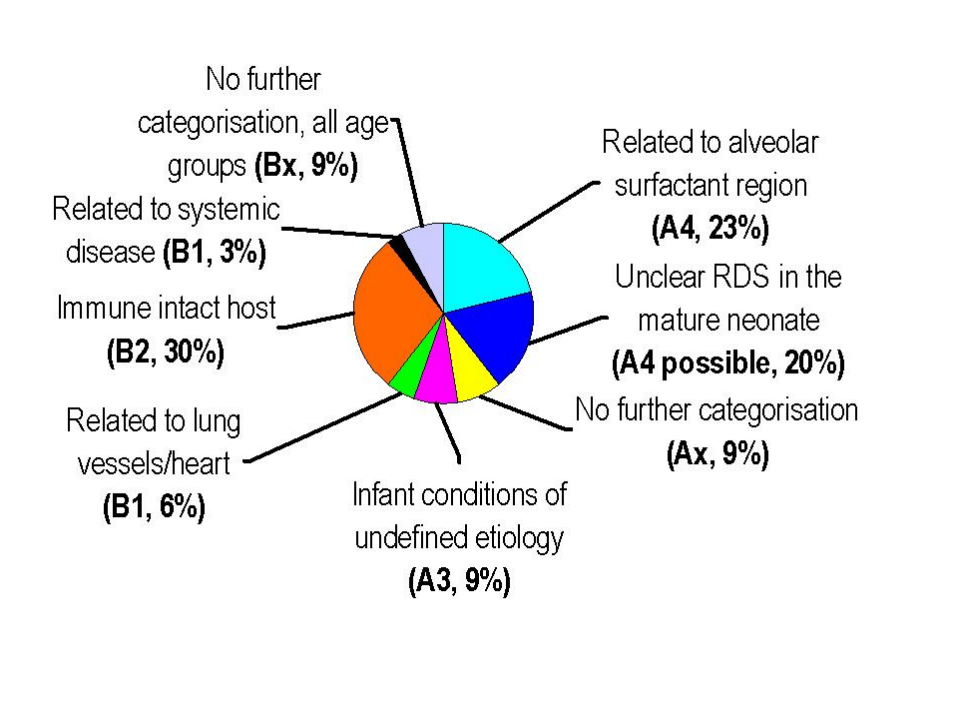
**The pie-chart illustrates the distribution of the 38 diagnoses classified according to a recent categorization system **[6]** expressed as percentages**.

Although all our cases were collected prospectively in 2005 and 2006 and inclusion was not restricted to patients with lung biopsy, the nomenclature of pediatric DPLD proposed in 2007 [6] was taken into account and all cases were attempted to categorize appropriately (Tables 1, 2 and 3, Figure 2).

Cases 1 to 21 were categorized into disorders more prevalent in infancy (A) (Tables 1 and 2). Patients 1-8 were clearly grouped into "related to the alveolar surfactant region" (A4) mainly based on lung biopsy or genetic findings. Patients 9 to 15 had DPLD manifesting in the neonatal period and likely involving the alveolar surfactant region; however definite diagnoses by biopsy were not obtained in these neonates. However it is possible that some of these patients might belong to categories A1 to A3. This could have only been clarified by lung biopsies, which were unfortunately not done. Similarly, patients 16 to 18 manifested their DPLD in infancy, but were only clinically defined by symptoms, radiologic, lung function and bronchoalveolar lavage findings typical for interstitial lung diseases. There were 3 cases (Patients 19-21) classified as "specific conditions of undefined etiology" (A3) and on the basis of biopsy in 2, as neuroendocrine hyperplasia of infancy. No cases with the diagnosis "alveolarization abnormalities" (A2) and "diffuse developmental disorders" (A1) were collected.

Cases 22 to 38 were categorized into disorders occurring at all ages (B) (Table 3). For the diagnosis of many of these entities a lung biopsy is not necessary. Indeed a biopsy was done in 5 cases and the other cases were categorized correctly with high confidence. Due to broadly exclusion of cases related to immunodeficiencies and transplantation (B3) and rheumatological or oncological diseases (B1), we had only one case "related to systemic diseases", i.e. biopsy proven sarcoidosis. The majority of subjects fell into the category "immune-intact hosts" (B2), i.e. were children with hypersensitivity pneumonitis and lastly children 36-38 were merely defined by symptoms, radiologic, lung function and bronchoalveolar lavage findings as typical for interstitial lung diseases, but not by lung biopsy.

### Diagnosis of monogenetic diseases

Genetic analysis may diagnose conditions related to the surfactant system (A4), e.g. as SP-B, SP-C, ABCA3 or GMCSF-receptor deficiencies or other conditions including immuno deficiencies with pulmonary manifestation. In six of the 15 cases known to be analysed for such genes aberrations were found, which lead to a definite molecular diagnosis in cases 1 to 5. Abnormalities were most frequently detected in ABCA3, followed by SP-C (Tables 1 and 2).

### Treatment and outcome

10 of the 12 patients with initial symptoms within the neonatal period were mechanically ventilated; of those who received exogenous surfactant the response was good in 60% of the cases (Table 6). In almost all children, systemic steroids were tried initially with a reported response rate of 90%. Of interest, in only two children with hypersensitivity pneumonitis allergen avoidance instead of steroids, i.e. the recommended treatment, were used (Table 7). Only in a minority of the patients with pediatric DPLD chloroquine was used (Tables 6 and 7).

**Table 6:** Treatment and outcome - Entities mainly prevalent in infants (A)

Patient No.	Mechanical ventilation	Surfactant treatment, response	O_2 _supply at rest	Immuno-suppressive treatment	Response to treatment	Long term out-come**
1	Yes*	yes; good	1 l/min	Systemic steroids	good but transient	died
2	Yes*	n.k.	n.k.	Systemic steroids; Azathioprin; Hydroxychloroquin	good; good; good	n.k.
3	Yes*	yes, good	100%	Systemic steroids	none	died
4	Yes*	yes, good	30-100%	Systemic steroids; Chloroquin	good; none	died
5	Yes	n.k.	2 l/min	Systemic steroids; Hydroxychloroquin	none; none	died
6	No	n.k.	2 l/min	Systemic steroids	n.k.	alive
7	No	n.k.	no	Systemic steroids	good	n.k.
8	n.k.	n.k.	4 l/min	Systemic steroids	n.k.	alive
9	Yes*	yes, none	80-100%	Systemic steroids	n.k.	died
10	Yes*	yes, none	transient	Systemic steroids	good	alive
11	Yes*	yes, good	1 l/min	Systemic steroids	good but transient	alive
12	Yes*	yes, good	transient	No steroids		alive
13	No	n.k.	n.k.	n.k.		n.k.
14	Yes*	yes, none	transient	No steroids		alive
15	No*	no	1-2 l/min	Systemic steroids	good	n.k.
16	No	no	n.k.	n.k.		n.k.
17	No*	n.k.		n.k.		n.k.
18	Yes*	n.k.		n.k.		n.k.
19	No	no	0,7-2 l/min	Systemic steroids	n.k.	alive
20	Yes	no	1 l/min	No steroids		alive
21	No	n.k.	2 l/min	Systemic steroids	good	alive

* Initial symptoms within the neonatal period; ** Follow up information was obtained one year after inclusion of the subject into the survey

n.k., not known;

**Table 7:** Treatment and outcome - Entities occurring at all ages (B), continues table 6

Patient No.	Mechanical ventilation	Surfactant treatment, response	O_2 _supply at rest	Immuno-suppressive treatment	Response to treatment	Long term out-come**
22	No	n.k.		n.k.		n.k.
23	No	n.k.		Systemic steroids	good	alive
24	No	no		Systemic steroids	good	n.k.
25	No	n.k.		Systemic steroids	good	alive
26	No	n.k.	no	Systemic steroids	good	alive
27	No	n.k.	0.5-1 l/min	Systemic steroids	good	alive
28	No	no		Systemic steroids	good	alive
29	No	no	1-2 l/min	Systemic steroids	good	alive
30	No	no	2 l/min	No steroids, allergen removal	good	alive
31	No	n.k.	no	No steroids, allergen removal	good	alive
32	No	no		Systemic steroids	good	alive
33	No	no		Systemic steroids	good	alive
34	No	n.k.		Systemic steroids	good	alive
35	No	n.k.		Systemic steroids	good	alive
36	No	no		Systemic steroids	good	n.k.
37	No	n.k.	0,3 l/min	n.k.		n.k.
38	n.k.	n.k.	no	n.k.		n.k.

* Initial symptoms within the neonatal period; ** Follow up information was obtained one year after inclusion of the subject into the survey

n.k., not known;

Of note, there was additional morbidity from concomitant diagnosis, including gastro-esophageal reflux, failure to thrive, and some auto-immune disorders which were treated separately (Tables 1, 2 and 3). Overall survival was 87% until the end of the observation period; however among infants with initial symptoms in the neonatal period, immediate survival was only 70% (Table 6).

### Role of the German consultation network for rare lung diseases

In Germany a working group on rare pediatric lung diseases is established and offers help with the diagnosis and management of cases (http://www.kids-lung-register.eu, http://www.kinderlungenregister.de, http://www.klreg.eu, http://www.ped-pneumology.de). About 45% of all cases reported through the German Surveillance Unit for Rare Paediatric Disorders (ESPED) were also managed in cooperation with the German consultation network (Tables 1, 2 and 3). Taken together, definite diagnoses and categorizations were achieved in 25 (cases 1-8 and 19-35) of the 38 cases, whereas in 13 cases this was not satisfactory. Of interest, among the latter cases in none a lung biopsy was done, only 3 were reported from University hospitals and in only 5 the German consultation network was involved.

## Discussion

This study assessed for the first time systematically the incidence and disease classification of pediatric DPLD in Germany, yielding a rate of 1.32 new cases per 1 million of children per year. Diseases caused by monogenetic defects most frequently manifested during infancy and in particular during the neonatal period, whereas those caused by exogenous factors occurred during later childhood and adolescence. The categorisation into a recently proposed classification system for pediatric DPLD was feasible, and demonstrated the need for a more thorough diagnostic effort in a number of cases. Guidance by the German consultation network for pediatric rare lung diseases facilitated a successful diagnosis and may also help in the future to improve treatment strategies.

The primary strength of the study is the use of an established German epidemiological surveillance system ESPED [7], which addressed all children's hospitals on a monthly basis with a very high and representative response rate. Furthermore, this is the first study to generate incidence data on the basis of a recent standardized classification system. The incidence estimate is a minimum since under-ascertainment is likely due to failure to diagnose DPLD or to report diagnosed cases. In order to minimize under-diagnosis all participating pediatric hospitals were given clear instructions regarding case definitions and diagnostic procedures before starting the surveillance. Assessment of potential under-reporting requires at least one additional reporting source. Unfortunately no such second reporting scheme is available for DPLD. In other studies underreporting in ESPED varied between 38 - 76% [7], with higher reporting rates for the more severe and chronic conditions such as metabolic diseases and diabetes. Among the 21 cases of pediatric DPLD occurring in infancy and categorized into "disorders more prevalent in infancy" (A), a satisfying diagnosis was established in 11. In 10 cases diagnostic efforts were stopped prematurely. Similar unresolved cases are less likely to be reported to the registry, whereas those with a definite diagnosis are more likely to be reported.

The categorization system applied here is based on a study that used lung tissue as the starting data point [6]. Therefore those cases that needed biopsy for diagnosis cannot be definitely categorized. Other differences are related to our case definition which excluded premature infants and children which cleared their disease within six weeks.

Recently the BPOLD registry, a web-based registry for rare (orphan) pediatric lung diseases in the United Kingdom, was established. In addition to interstitial lung diseases six other rare lung conditions were collected [8]. The advantage of the system is a rapid and direct communication between the involved sites. This may allow addressing the general problem that many of the pediatric DPLD are initially diagnosed and treated in regional hospitals, as can be seen by the rate of 50% of all cases reported in our study from regional hospitals. A disadvantage is that of the 15 cases of interstitial lung disease reported over a one year period only one case was confirmed. It is of importance to offer assistance in the management of these rare diseases. This is feasible by advice on genetic testing which was done in our study in 6 out of the 13 cases not satisfactory diagnosed initially.

The current study clearly showed that pediatric DPLD represent orphan diseases, which need to be more carefully addressed by both, clinicians and policymakers, health regulators and government. This is particularly relevant in the diseases occurring in infants, as the mortality is high (Table 6). Appropriate diagnostic classification and categorization is of great importance for indicating a correct prognosis and treatment in DPLD. The clinicians responsible have to be very insistent as often expensive genetic tests or relatively invasive procedures (open lung biopsy), which they are not familiar with on a routine basis, have to be performed adequately. Similarly, the right therapeutic measures need to be systematically installed and supervised. Such tasks should be done in close cooperation with or in an experienced centre. Web-based communication and consultation systems may facilitate the management of these patients. Their implementation will allow easy access to reference pediatric pathologists, radiologists and other experts. Lastly, the pseudonymised collection of rare cases, best in association with bio-banked materials, and the international integration of national networks, will allow a more rapid progress for patients with these cumbersome illnesses.

The knowledge of a causal mutation and, if sufficient numbers of similar cases are collected, the possibility of a reliable prediction of response to therapy and the prognosis, are important from an individual perspective, as uncertainty causes significant stress and anxiety for many patients and their families [9]. For instance in patient 1, bearing lethal SP-B mutations where therapeutic interventions like steroids or whole lung lavages are known to be ineffective [10], unnecessary treatments can be avoided. Among the mutations in genes leading to pediatric DPLD related to the alveolar surfactant region, ABCA3 mutations were the most frequent ones (Table 1). Based on these data and those obtained previously [11-13], the diagnostic workup of neonates and infants with suspected DPLD, a non-resolving course and exclusion of other causes, should include initial genetic testing for SFTPB and SFTPC as these are rather small genes, followed by analysis of the large ABCA3 gene. If genetic testing remains negative, lung biopsy is the next consequent step. In older children lung biopsy may be done prior to the genetic tests, since by this strategy several other entities which cannot be diagnosed genetically yet, might be diagnosed. We found that all ABCA3 mutations identified were different from each other and not described previously; they will be the topic of a separate future report.

## Conclusions

We present the incident cases of pediatric DPLD in Germany over a two year period and demonstrate that particularly those cases which were assessed with the help of the German consultation network for pediatric rare lung diseases, could definitely be diagnosed and categorized into an advanced classification system. We suggest that systematic surveys, disease classification and clinical management systems represent a critical prerequisite for future improvements of treatment and prognosis in the rare children with DPLD.

## Abbreviations

DPLD: Diffuse parenchymal lung diseases; ESPED: Erhebungseinheit für seltene pädiatrische Erkrankungen in Deutschland.

## Competing interests

The authors declare that they have no competing interests.

## Authors' contributions

GM designed the study, participated in and lead the categorization of the subjects, and wrote the manuscript, HM and HD collected, categorized and organized the data, FA, ZT, LP, and BF discussed and verified the cases, HD and KR organized and supervised the performance of the study instrument. All authors participated in drafting the manuscript and approved the final manuscript.
